# Mild Zellweger syndrome due to functionally confirmed novel *PEX1* variants

**DOI:** 10.1007/s13353-019-00523-w

**Published:** 2019-10-18

**Authors:** Patryk Lipiński, Piotr Stawiński, Małgorzata Rydzanicz, Maria Wypchło, Rafał Płoski, Teresa Joanna Stradomska, Elżbieta Jurkiewicz, Sacha Ferdinandusse, Ronald J. A. Wanders, Frederic M. Vaz, Anna Tylki-Szymańska

**Affiliations:** 1 Department of Pediatrics, Nutrition and Metabolic Disease, The Children’s Memorial Health Institute, Al. Dzieci Polskich 20, 04-730 Warsaw, Poland; 2 Department of Medical Genetics, Medical University of Warsaw, Warsaw, Poland; 3 Postgraduate School of Molecular Medicine, Medical University of Warsaw, Warsaw, Poland; 4 Department of Biochemistry, Radioimmunology and Experimental Medicine, The Children’s Memorial Health Institute, Warsaw, Poland; 5 Department of Diagnostic Imaging, The Children’s Memorial Health Institute, Warsaw, Poland; 6 Laboratory Genetic Metabolic Diseases, Academic Medical Center, University of Amsterdam, Amsterdam, The Netherlands

**Keywords:** Zellweger spectrum disorders, *PEX1* gene, C26:0-lysoPC

## Abstract

Zellweger spectrum disorders (ZSD) constitute a group of rare autosomal recessive disorders characterized by a defect in peroxisome biogenesis due to mutations in one of 13 *PEX* genes. The broad clinical heterogeneity especially in late-onset presenting patients and a mild phenotype complicates and delays the diagnostic process. Here, we report a case of mild ZSD, due to novel *PEX1* variants. The patient presented with an early hearing loss, bilateral cataracts, and leukodystrophy on magnetic resonance (MR) images. Normal results of serum very-long-chain fatty acids (VLCFA) and phytanic acid were found. Molecular diagnostics were performed to uncover the etiology of the clinical phenotype. Using whole exome sequencing, there have been found two variants in the *PEX1* gene—c.3450T>A (p.Cys1150*) and c.1769T>C (p.Leu590Pro)*.* VLCFA measurement in skin fibroblasts and C26:0-lysoPC in dried blood spot therefore was performed. Both results were in line with the diagnosis of ZSD. To conclude, normal results of routine serum VLCFA and branched-chain fatty acid measurement do not exclude mild forms of ZSD. The investigation of C26:0-lysoPC should be included in the diagnostic work-up in patients with cataract, hearing loss, and leukodystrophy on MR images suspected to suffer from ZSD.

## Introduction

Zellweger spectrum disorders constitute a group of rare autosomal recessive disorders characterized by a defect in peroxisome biogenesis due to mutations in one of 13 *PEX* genes (Klouwer et al. 2015). The broad clinical heterogeneity especially in late-onset presenting patients and a mild phenotype complicates and delays the diagnostic process (Klouwer et al. 2016; Berendse et al. 2016).

Here, we report a patient diagnosed with ZSD at the age of 21 presenting with early hearing loss and then bilateral cataracts and leukodystrophy with normal results of standard biochemical markers (including serum very-long-chain fatty acids (VLCFA)), in whom the molecular analysis (whole exome sequencing (WES)) prompted to investigation of C26:0-lysoPC in DBS and VLCFA in skin fibroblasts to confirm the diagnosis.

## Case report

The patient was the third child of nonconsanguineous Polish parents, born at term from an uncomplicated pregnancy with a body mass of 3100 g. During the first year of life, he had undergone two episodes of pneumonia; during the first episode, a vitamin K–dependent coagulopathy was diagnosed. The laboratory analyses revealed anemia and slightly elevated liver transaminases (Table 1). At the age of 1.5 years, nystagmus was noted. Liver transaminases had normalized but the prothrombin time was still prolonged.

**Table 1:** The patient’s signs and symptoms and biochemical and molecular outcome

Age	Symptoms and signs	Results of laboratory tests/imaging procedures
First year of life	2 episodes of pneumonia; vitamin K–dependent coagulopathy	Elevated liver transaminases (AST 109,74; ALT 65,75), prolonged PT 17.2/15.4, normal results of metabolic screening tests^#^
1.5 y	Third episode of pneumonia; nystagmus	Normal liver transaminases, prolonged PT 18.5, normal results of metabolic screening tests
4 y	Delayed psychomotor development; hearing loss	n.a.
12 y	Partial wheelchair dependency due to spastic paresis; mild intellectual disability; slowing head circumference growth	n.a.
18 y	Bilateral cataracts	Normal liver transaminases, normal PT, normal results of metabolic screening tests, CT imaging—mild generalized cortical and subcortical atrophy
21 y	Total wheelchair dependency; anger attacks with progressive mental retardation; microcephaly	Normal results of metabolic screening tests, *C24:0/C22 0–0.910; *N* < 0.96 C26:0/C22 0–0.015; *N* < 0.02 Phytanic acid (μg/mL)—0.37; *N* < 3.3 **C24:0/C22 0–0.940; *N* < 0.96 C26:0/C22 0–0.020; *N* < 0.02 Phytanic acid (μg/mL)—0.47; *N* < 3.3 VLCFA in skin fibroblasts C24:0/C22 0–4.09; *N* = 1.68–2.92 C26:0/C22 0–0.92; *N* = 0.03–0.10 C26:0-lysoPC (pmol/mg protein)—131; *N* = 2–14 Leukodystrophy on MR imaging (see Fig. 1)

*mo* months, *y* years, *n.a*. not analyzed, *CT* computed tomography, *MR* magnetic resonance, *ALT* alanine aminotransferase (U/l), *AST* aspartate aminotransferase (U/l), *PT* prothrombin time (s)

Reference values: ALT, < 18 months, < 55/60 U/L; 18 months–12 years, boys, < 40 U/L, girls, < 35 U/L; > 12 years, boys, < 26 U/L, girls, < 22 U/L; AST, < 52 U/L

*1st measurement

**2nd measurement

^#^GC/MS analysis of organic acids in urine, tandem mass spectrometry analysis (MS/MS), transferrin isoforms analysis, serum ammonia and lactate, serum VLCFA, urine and serum arabitol, urinary glycosaminoglycans analysis

The patient’s psychomotor development was delayed; he was able to walk alone at the age of 3.5 years. From the age of 5 years, he received a hearing aid because of hypoacusis. At the age of 12 years, a progressive spastic paresis was diagnosed. A mild intellectual disability and slowing head circumference growth were also observed. At the age of 18, bilateral cataracts were noted. CT brain imaging done at that age revealed mild generalized cortical and subcortical atrophy.

The family history revealed that patient’s older sister had presented with similar signs including more pronounced intellectual disability, bilateral hearing loss, and cataracts but no diagnosis was established.

**Fig. 1:**
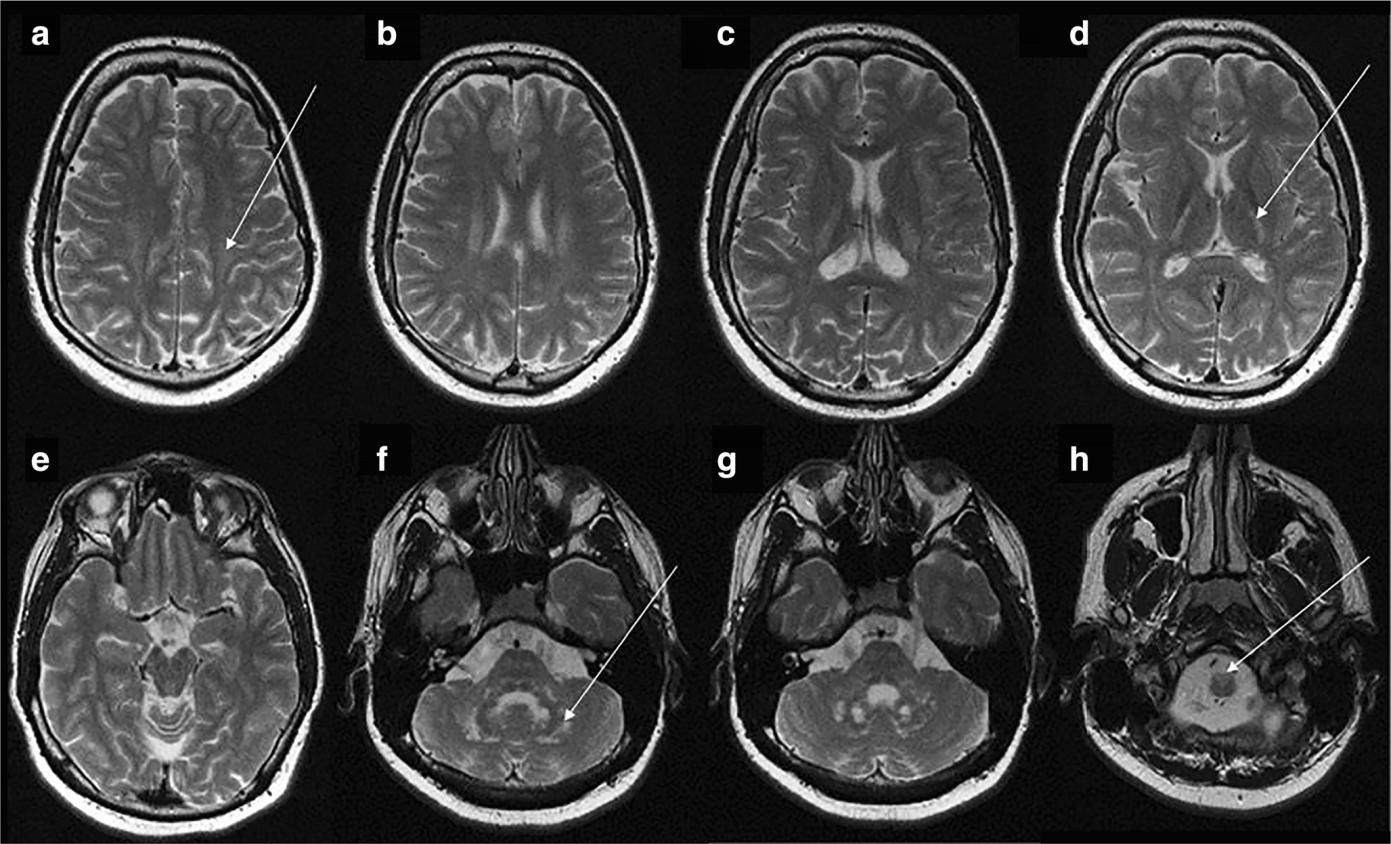
MRI examination at the age of 21 years. Axial T2-weighted images. See the bilateral hyperintense signal of the posterior part of the centrum semiovale (arrow on **a**), posterior limb of internal capsule (arrow on **d**), cerebellar white matter (arrow on **f**). The antero-medial part of medulla oblongata is also hyperintesive (arrow on **h**)

The patient became completely wheelchair dependence when at 21 years old. Due to progressive mental retardation, magnetic resonance (MR) imaging was done and the brain T2-weighted images (see Fig. 1) showed cerebral white matter hyperintensity in the both centrum semiovale posteriorly with sparing of subcortical fibers, bilateral areas of signal increase surrounding posterior parts of lateral ventricles, hyperintensive posterior limbs of the internal capsules, and the small hyperintense signal in the genu and splenium of the corpus callosum. Infratentorially, the signal increased in the central cerebellar white matter area and in the hilus of the dentate nucleus, bilaterally. Dentate nucleus between hilus and central cerebellar white matter were spared. Hyperintensity of the anteriomedial part of the medulla oblongata was also seen.

There were no clinical signs of adrenal insufficiency. He had no dysmorphic features except microcephaly, which was noted until the age of 21 years old.

**Fig. 2:**
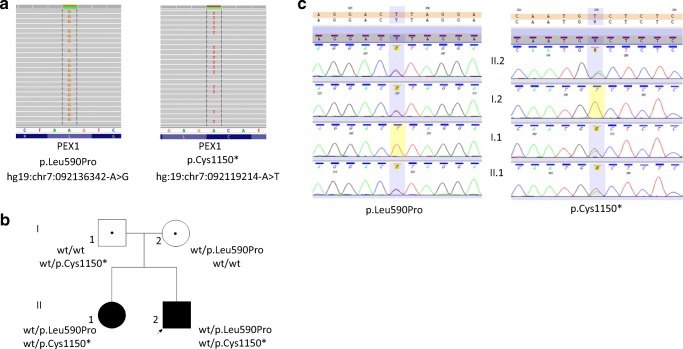
**a** WES results in the proband—view from Integrative Genomics Viewer (IGV). **b** Pedigree of the family with phenotype/genotype information. The proband is indicated with black arrow (wt wild type). **c** Graphical presentation of Sanger sequencing verification of detected *PEX1* variants in the proband and her family—view from Variant Reporter 1.1

Molecular diagnostics were performed to uncover the etiology of the clinical phenotype. Venous blood samples were collected from the proband and his three family members (father, mother, and older sister). WES was performed in the proband DNA sample using SureSelect Target Enrichment kit All Exon v5 (Agilent, Agilent Technologies, Santa Clara, CA) on HiSeq 1500 (Illumina, San Diego, CA). The mean depth of coverage in sequenced sample was 86×; 94% of target sequence was covered min 20× and 98% min 10×. Raw data was analyzed as previously described (Ploski et al. 2014). Using WES, there have been found two variants in the *PEX1gene* (NM_000466.2)—c.3450T>A (p.Cys1150*) and c.1769T>C (p.Leu590Pro, Fig. 2). Sanger sequencing showed presence of heterozygous *PEX1* p.Cys1150* in the proband, his affected sister, and their father whereas the p.Leu590Pro variant was found in the proband, his affected sister, and their mother. The p.Cys1150* variant is likely to be damaging since it introduces premature stop codon predicted to truncate the PEX1 protein. The p.(Leu590Pro) variant is predicted to be damaging by MetaSVM (a bioinformatic tool ntegrating prediction scores from SIFT, PolyPhen-2, GERP++, MutationTaster, Mutation Assessor, FATHMM, LRT, SiPhy, and PhyloP) (https://varsome.com). Both variants have not been reported in GnomAD (http://gnomad.broadinstitute.org) or in our own database including over 1000 WES samples from a Polish population.

Thus, based on WES analysis, we found two novel potentially damaging *PEX1* variants which occurred in trans in both affected sibs consistent with autosomal recessive inheritance.

In order to determine whether these variants had affected enzymatic function, biochemical examinations were performed as described elsewhere (Stradomska and Tylki-Szymańska 1996; Takemoto et al. 2003). Surprisingly, we found normal results of serum VLCFA and phytanic acid (Table 1). We therefore continued and performed VLCFA measurement in skin fibroblasts and C26:0-lysoPC in dried blood spot as described by Klouwer et al. (2017) in the Laboratory of Genetic Metabolic Diseases in Amsterdam. Both results (Table 1) were in line with the diagnosis of ZSD.

## Discussion

The paper presents diagnostic difficulties in establishing a diagnosis of ZSD in a 21-year-old patient based on standard biochemical markers, including serum VLCFA and phytanic acid.

Nowadays, next-generation sequencing techniques including gene panels or event whole exome sequencing are new possibilities to identify the molecular background of diseases with an unknown etiology. Because of this, there are an increasing number of patients with a mild phenotype of ZSD initially diagnosed by molecular analysis (Ebberink et al. 2010; Ebberink et al. 2012; Sevin et al. 2011; Ventura et al. 2016; Rydzanicz et al. 2017). The molecular results, however, should always be interpreted in combination with the clinical phenotype and appropriate laboratory analyses. The presented case and also findings from other reports supports the thesis that the level of serum markers of peroxisome dysfunction may not reflect the level of tissue accumulation (Berendse et al. 2016; Sevin et al. 2011). As in our previous report, VLCFA levels in patients presenting with a mild phenotype are generally below the values characteristic for patients with classical severe disease (Rydzanicz et al. 2017).

The standard biochemical serum markers of peroxisomes dysfunction include accumulation of VLCFA, phytanic and pristanic acid, and C27-bile acid intermediates and a deficiency of erythrocyte plasmalogens (Stradomska and Tylki-Szymańska 2009; Wanders et al. 2017). A positive correlation of C26:0 and C26:0/C22:0 ratio with VLCFA accumulation has been shown (Berendse et al. 2016; Stradomska and Tylki-Szymańska 2009; Raas-Rothschild et al. 2002; Tran et al. 2014); however, in some patients, especially those with a mild phenotype or affected with certain *PEX* genes mutations, serum VLCFA can be completely normal (Berendse et al. 2016; Ebberink et al. 2010; Sevin et al. 2011). Normalization of serum VLCFA with age has been also reported (Berendse et al. 2016). Therefore, there is a need of better biochemical markers in diagnostic work-up in patients suspected to suffer from ZSD. Recently, Klouwer et al. commenced a novel serum biomarker, C26:0-lysoPC, and it has been reported as a sensitive marker in ZSD patients, more sensitive than other biochemical markers such as serum VLCFA and phytanic acid (Klouwer et al. 2017). Yet, even if all test results are normal in plasma and urine, but a high level of suspicion for a ZSD remains, additional testing in fibroblasts should be considered to definitely rule out a peroxisomal disorder (Klouwer et al. 2016).

Based on the occurrence of early hearing loss and then bilateral cataracts and leukodystrophy on MR images, we could retrospectively categorize patient to be suffered from mild ZSD based on the severity scoring scale proposed by Klouwer et al. (Klouwer et al. 2018). As in our previously published case and report of Weller et al. (Weller et al. 2008), the leukoencephalopathy and brain atrophy are the commonest changes found in mildly affected patients. This case supports also the hypothesis termed by Klouwer et al. about eye abnormalities (especially cataract) and hearing loss as the key symptoms in ZSD.

## Conclusions

Normal results of routine serum VLCFA and branched-chain fatty acid measurement do not exclude mild forms of ZSD.

The investigation of C26:0-lysoPC should be included in the diagnostic work-up in patients with hearing loss, cataract, progressive intellectual disability, spastic paraparesis, and leukodystrophy on MR images suspected to suffer from ZSD.

## Abbreviations

C26:0-lysoPC: C26:0-Lysophosphatidylcholine
CT: Computed tomography
DBS: Dried blood spot
MR: Magnetic resonance
NGS: Next-generation sequencing
VLCFA: Very-long-chain fatty acids
WES: Whole exome sequencing
ZSD: Zellweger spectrum disorders
